# Dissecting the biophysical mechanisms of oleate hydratase association with membranes

**DOI:** 10.3389/fmolb.2024.1504373

**Published:** 2025-01-08

**Authors:** William A. Lathram, Robert J. Neff, Ashley N. Zalla, James D. Brien, Vivekanandan Subramanian, Christopher D. Radka

**Affiliations:** ^1^ Department of Microbiology, Immunology, and Molecular Genetics, University of Kentucky, Lexington, KY, United States; ^2^ Department of Pharmaceutical Sciences, University of Kentucky, Lexington, KY, United States

**Keywords:** oleate hydratase (OhyA), phospholipid, membrane bilayer, fluorescence correlation spectroscopy (FCS), phosphorus nuclear magnetic resonance (^31^P NMR), membrane binding, lipid-protein interaction

## Abstract

This study investigates the dynamics of oleate hydratase (OhyA), a bacterial flavoenzyme from *Staphylococcus aureus*, and its interactions with lipid membranes, focusing on the factors influencing membrane binding and oligomerization. OhyA catalyzes the hydration of unsaturated fatty acids, playing a key role in bacterial pathogenesis by neutralizing host antimicrobial fatty acids. OhyA binds the membrane bilayer to access membrane-embedded substrates for catalysis, and structural studies have revealed that OhyA forms oligomers on membrane surfaces, stabilized by both protein-protein and protein-lipid interactions. Using fluorescence correlation spectroscopy (FCS), we examined the effects of membrane curvature and lipid availability on OhyA binding to phosphatidylglycerol unilamellar vesicles. Our results reveal that OhyA preferentially binds to vesicles with moderate curvature, while the presence of substrate fatty acids slightly enhanced the overall interaction despite reducing the binding affinity by 3- to 4-fold. Complementary phosphorus-31 (^31^P) NMR spectroscopy further demonstrated two distinct binding modes: a fast-exchange interaction at lower protein concentrations and a longer lasting interaction at higher protein concentrations, likely reflecting cooperative oligomerization. These findings highlight the reversible, non-stoichiometric nature of OhyA•membrane interactions, with dynamic binding behaviors influenced by protein concentration and lipid environment. This research provides new insights into the dynamic behavior of OhyA on bacterial membranes, highlighting that initial interactions are driven by lipid-mediated protein binding, while sustained interactions are primarily governed by the protein:lipid molar ratio rather than the formation of new, specific lipid-protein interactions. These findings advance our understanding of the biophysical principles underlying OhyA’s role in bacterial membrane function and virulence.

## 1 Introduction

Membrane-targeting domains are essential for recruiting signaling molecules to membranes, and protein association with lipid membranes often occurs in response to extracellular or intracellular stimuli. However, these interactions are typically transient, allowing for precise temporal regulation of signaling pathways (Campagnola et al., 2015). The dynamic nature of these interactions also affects how proteins locate their target substrates, though the specifics of this process are not well understood. Peripheral membrane proteins play critical roles in key cellular processes such as signaling, cell division, and vesicle trafficking (Munro, 2002; DiNitto et al., 2003; Cho and Stahelin, 2005). These proteins associate with lipid membranes through various mechanisms, including lipid modifications and membrane-targeting domains. Their membrane binding sites can interact directly with specific lipid molecules, outer head groups (Stahelin, 2009), or the internal hydrocarbon backbone (Mulgrew-Nesbitt et al., 2006; Lomize et al., 2007; Murphy et al., 2009). Quantitative studies of protein-membrane affinity are crucial for unraveling the mechanics of these associations, understanding how the biophysical properties of membranes influence interactions, and determining the energetics of protein binding and insertion.

Oleate hydratase (OhyA) is a bacterial flavoenzyme that catalyzes water addition to *cis* double bonds of unsaturated fatty acids, producing hydroxylated fatty acids (*h*FA) (Radka et al., 2021b; Radka et al., 2024). OhyA activity facilitates a crucial step in the reduction of linoleic acid in commensal gut bacteria (Yang et al., 2017), and as a biocatalyst in the production of *h*FA intermediates (Prem et al., 2022). Commensal bacterial OhyA utilizes host unsaturated fatty acids to produce *h*FAs that work to reduce gut inflammation and bacterial membrane destabilization (Galbraith et al., 1971; Greenway and Dyke, 1979; Raychowdhury et al., 1985; Zheng et al., 2005; Miyamoto et al., 2015; Saika et al., 2019). Recent studies have displayed the role of OhyA as a virulence determinant in *Staphylococcus aureus*, a common human pathogen, and the primary cause of skin infection (Chalmers and Wylam, 2020). The initial response from the innate immune system, during a *S. aureus* infection, is for host cells to secrete antimicrobial peptides and fatty acids (Liu et al., 2018). To combat this response, *S. aureus* expresses OhyA to detoxify antimicrobial fatty acids in the host (Subramanian et al., 2019). A murine skin infection model has observed that OhyA activity represses the immune response, while OhyA disruption compromised *S. aureus* virulence (Radka et al., 2021a).

**FIGURE 1:**
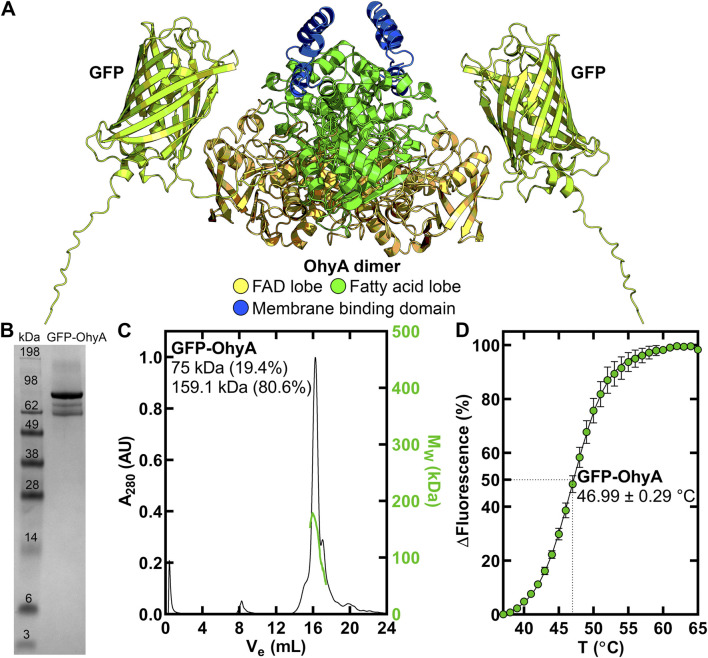
Purification and thermal stability of GFP-OhyA. **(A)**, AlphaFold-generated dimer model of GFP-OhyA, with colors representing different functional domains. **(B)**, GFP-OhyA appears as a 97 kDa monomer, with a purity of >95% as confirmed by denaturing gel electrophoresis. The theoretical molecular weight of the GFP-OhyA monomer is 96.8 kDa. **(C)**, Gel filtration chromatogram (*black*) of GFP-OhyA, eluted from a Superose 6 Increase 10/300 GL column, overlaid with the molar mass distribution (*green*) from multi-angle light scattering (SEC-MALS). According to SEC-MALS, GFP-OhyA primarily exists as a 159.1 kDa dimer. **(D)**, Thermal denaturation analysis of GFP-OhyA was performed to assess structural integrity of the protein. Assays (N = 5) were conducted with 1 mg/mL GFP-OhyA, and the data were fitted to the Boltzmann equation. The mean melting temperature is reported with ± S.D.

We study the *S. aureus* OhyA as a representative homolog (Robert et al., 2024). OhyA forms a homodimer in solution comprised of three key functional domains (Figure 1A): fatty acid lobe to bind unsaturated fatty acid substrate, FAD-binding lobe to bind coenzyme FAD, and carboxy terminus made of amphipathic helices that bind the membrane bilayer to interact with unsaturated fatty acid substrate (Radka et al., 2021b; Radka et al., 2024). The current model is the carboxy terminus transfers fatty acid from the membrane to the active site through a hydrophobic tunnel. Lipid binding studies demonstrated OhyA is a peripheral membrane-associated protein that directly interacts with the bilayer through surface electrostatic interactions between its carboxy terminus and the phosphate layer of the membrane (Radka et al., 2024). Structural analysis of an OhyA•membrane complex by cryo-electron microscopy showed OhyA assembles into higher order oligomers on the membrane surface, and the oligomers are stabilized by intermolecular interactions between adjacent OhyA dimers (Oldham et al., 2024). Thus, protein-protein and protein-lipid interactions both drive OhyA•membrane complex formation, but decomposition of their contributions to binding has not been investigated.

Fluorescence correlation spectroscopy (FCS) is a powerful tool for rapid equilibrium analysis of fluorescent molecules and has been used to study the dynamics of protein-membrane binding interactions (Rhoades et al., 2006; Thomas et al., 2015; Kruger et al., 2017; Vesga et al., 2023), membrane toxin conformational alterations, and elucidate membrane-receptor binding (Chen et al., 2009; Harwardt et al., 2018; Ponmalar et al., 2019). FCS is a highly sensitive technique that measures the intensity fluctuation emitted by single molecules passing in and out of a focused light, yielding intensity fluctuations (Elson and Magde, 1974; Elson, 2011; Elson, 2013). The intensity fluctuations correspond to variations in the number of molecules interacting within, and diffusing through, the confocal plane (Elson and Magde, 1974). Evaluation of the decay of the intensity fluctuations enables the determination of diffusion coefficients and binding rate constants. Additionally, spectroscopic techniques detect signals from both aqueous and lipid phases simultaneously, eliminating the need for physical separation of these phases, which could disrupt equilibrium (Santos et al., 2003).

OhyA has shown a nanomolar binding affinity to phosphatidylglycerol, the dominant anionic phospholipid in the *S. aureus* membrane (Kuhn et al., 2015), in previous surface plasmon resonance (SPR) experiments (Radka et al., 2024). However, these experiments used large unilamellar vesicles (LUVs) composed of both phosphatidylglycerol and phosphatidylcholine due to the L1 sensor chip’s inefficiency in capturing anionic phospholipids. Moreover, SPR only allowed titration of one component (protein), limiting the analysis. In contrast, FCS does not require immobilization of components, allowing for experimental designs with varying concentrations of proteins and/or lipids and providing greater flexibility in the types of molecules that can be studied.

In this study, we employed a green fluorescent protein (GFP)-OhyA chimeric protein in FCS experiments to investigate the dynamics of OhyA binding phosphatidylglycerol unilamellar vesicles, focusing on the effects of membrane curvature and substrate availability on OhyA•membrane interactions. Our results revealed that GFP-OhyA shows a moderate preference for binding to LUVs over small unilamellar vesicles (SUVs), highlighting the role of membrane curvature in driving the interaction. Incorporating the 18:1Δ9 unsaturated fatty acid substrate into vesicles had only a minor impact on OhyA•membrane binding, reinforcing that curvature is the primary factor influencing membrane association. Titration experiments with varying protein and lipid concentrations identified two distinct binding modes: as protein concentration increased, OhyA accumulated on protein-loaded vesicles, whereas increasing the concentration of accessible lipids decreased the protein occupancy on individual vesicles. These findings offer new insights into the dynamic behavior of OhyA on membrane surfaces and suggest that protein distribution on membranes can be highly uneven due to non-stoichiometric interactions.

## 2 Materials and methods

### 2.1 Plasmid construction for GFP-OhyA expression in *E. coli*

*The gfp-ohyA* gene was constructed to contain an amino terminal His_6_ tag followed by the *gfp* gene that was linked by a flexible linker (amino acid sequence GGGGSGGSS) to *ohyA* as previously described (Radka et al., 2024). The initiating methionine residues of GFP and OhyA were mutated to alanine residues to eliminate alternative transcription start sites during overexpression. Thus, the open reading frame encoded fMet-His_6_ tag-GFP-flexible linker-OhyA. The *gfp-ohyA* gene was cloned into the pET28a 5′-NcoI restriction site using the Gibson Assembly method to make pGFP-OhyA. Overexpression of *gfp-ohyA* is driven by the strong bacteriophage T7 promoter in this plasmid.

### 2.2 Preparation of GFP-OhyA

The pGFP-OhyA plasmid was transformed into *Escherichia coli* BL21 (DE3) cells, and isolates were selected on Luria broth agar plates containing 50 μg/μL kanamycin (Gold Biotechnology). The transformants were cultured in Luria broth with 50 μg/μL kanamycin at 37°C with shaking at 200 rpm. Once the cells reached an OD_600_ of 0.6, the temperature was lowered to 16°C, and protein expression was induced overnight with 1 mM isopropyl-β-D-thiogalactoside (Gold Biotechnology). After induction, the cells were harvested and lysed in a buffer containing 20 mM Tris, pH 8.0, 10 mM imidazole, 200 mM NaCl. The GFP-OhyA protein was purified from the lysate using nickel agarose beads (Gold Biotechnology) and eluted with a buffer containing 20 mM Tris, pH 8.0, 250 mM imidazole, and 200 mM NaCl. The eluted protein was further purified by gel filtration using a HiLoad Superdex 200 16/60 column (Cytiva Life Sciences) into a buffer with 20 mM Tris, pH 8.0, and 200 mM NaCl. The molecular weight of GFP-OhyA was estimated by SEC-MALS using a Superose 6 Increase 10/300 GL column (Cytiva Life Sciences) with three detectors connected in series: an Agilent 1200 UV detector (Agilent Technologies), a Wyatt DAN HELEOS II multi-angle light-scattering and a Wyatt Optilab T-rEX differential refractive index detector (Wyatt Technologies). The column was equilibrated in 20 mM Tris, 200 mM NaCl, pH 7.6, and 100 μL of GFP-OhyA (2 mg/mL) was injected at a flow rate of 0.5 mL/min at 25°C. Data were recorded and analyzed with the Wyatt Astra software (version 8), and plotted as a molar mass distribution superimposed on a chromatogram of A_280_ versus elution volume.

The thermal stability of GFP-OhyA was determined by a Sypro Orange-based fluorescence assay (Huynh and Partch, 2015). Solutions (30 μL) of GFP-OhyA (1 mg/mL) in 50 mM K_2_HPO_4_, 150 mM NaCl, pH 6, and 2.5 × Sypro Orange dye were added to the wells of a ThermoGrid optically clear PCR plate (Denville Scientific). The plates were centrifuged at 1,000 × *g* for 5 min and then analyzed by the ABI 7300 real-time PCR system as described previously (Radka et al., 2020). The temperature was ramped from 25°C to 95°C at 1°C/min with the fluorescence read six times at each temperature ramp. The resulting data were fitted to a Boltzmann sigmoidal equation to determine the melting point. The experiment was repeated five times, the thermal melting temperature of each replicate was determined independently, and the melting points from each replicate were averaged to determine the reported thermal melting point.

### 2.3 Unilamellar vesicle production

Charged unilamellar vesicles were prepared from three different lipid compositions: 100% anionic dioleoylphosphatidylglycerol (DOPG) (Avanti Polar Lipids), cationic dioleoylphosphatidylcholine (DOPC) (Avanti Polar Lipids), and a 99:1 mixture of DOPG and 18:1Δ9 (Ambeed) (DOPG/OA). For 100% DOPG or 100% DOPC vesicles, 60 μg of lipid was dissolved in chloroform and added to a 16 mm glass culture tube. For the DOPG/OA vesicles, 60 μg of DOPG and 2.12 μg of 18:1Δ9 were added to the glass culture tube. Micelles were prepared from 60 μg neutral triolein (Avanti Polar Lipids). The chloroform was evaporated under nitrogen gas using the Reacti-VapIII (Thermo Scientific, #TS-18826). The dried lipids were resuspended to a final concentration of 3 mM lipids in 50 mM KPO_4_, 150 mM NaCl, pH 6.0 and sonicated at 37^∘^C for at 5–10 min. Lipids were then manually extruded back and forth 75 times using a 10 mm filter support (Avanti Polar Lipids) fitted with either 0.03 μm or 0.1 μm polycarbonate membranes to produce SUVs or LUVs, respectively. The 0.1 μm polycarbonate membrane was used to prepare micelles. The hydrodynamic size and polydispersity of each lipid particle were measured using dynamic light scattering (DLS) with the RNA-LNP application of a Stunner instrument (Unchained Labs). Measurements were conducted under default settings: a 142° scattering angle, a 660 nm laser, and four 1 s acquisitions with automatic angle selection and outlier exclusion. At a lipid concentration of 3 μM, DLS confirmed that lipid particles remained monodisperse and did not aggregate, even at the highest lipid concentration used in the binding titration.

To fluorescently label the vesicles or micelles for FCS standardization, 9.76 μL of a 1:100 dilution from a 1 mg/mL 18:1 Liss Rhod PE (Avanti Polar Lipids) stock solution was added to each lipid mixture in a 16 mm glass culture tube. The chloroform was evaporated under nitrogen gas, and the vesicles or micelles were then assembled as described above. This process produced Rhod PE-labeled SUVs, LUVs, and micelles for assessing free diffusion.

### 2.4 Binding titrations

To determine the impact of protein concentration on binding, 0.014, 0.028, 0.056, 0.113, 0.22, 0.45, 0.9, 1.8 μM GFP-OhyA was mixed with 0.188 μM accessible lipid. To determine the impact of accessible lipid concentration on binding, 0.0115, 0.023, 0.047, 0.094, 0.188, 0.375, 0.75, 1.5, 3 μM lipid was mixed with 0.113 μM GFP-OhyA. The samples were 50 μL in volume and incubated in 50 mM KPO_4_, 150 mM NaCl, pH 6.0 for 5 m at 20°C before FCS data acquisition. The samples used for FCS standardization were 0.113 μM GFP-OhyA and 0.188 μM Rhod PE-labeled DOPC LUVs, DOPG SUVs and LUVs, DOPG/OA SUVs and LUVs, and triolein micelles.

### 2.5 FCS set-up

FCS measurements were conducted using a custom-built microscope within the University of Kentucky Microscopy Core. Approximately 50 μL of freshly prepared, fluorescently labeled sample was placed onto an 18 × 18 mm Zeiss no. 1.5 coverslip, positioned inside a 35 mm Petri dish with an 18 mm hole (Cell E&G, Cat #: PDH00002-200). The Petri dish was mounted on a Nikon Eclipse Ti2 microscope equipped with a PicoQuant PicoHarp 300 Time-Correlated Single Photon Counting (TCSPC) system. A 532 nm laser, set at 1% power (29 μW), was used to excite the fluorescent labels, and a ×60 water immersion objective focused the laser beam on the sample. Photon detection was handled by the PicoHarp 300 TCSPC module. Each FCS result represents the average of three 60 s measurements. To avoid background interference from immobilized molecules on the coverslip, all measurements were taken at least 30 μm above the glass surface. Raw data was processed using SymPhoTime 64 (PicoQuant) software.

Laser power and pinhole diameter were calibrated using highly diluted GFP-OhyA and Rhod PE solutions, excited at 488 nm and 563 nm, respectively. At least five autocorrelation functions were obtained at 20°C for each condition, adjusting the laser irradiance to achieve consistent molecular brightness at 1% power. The measured laser power was 29 μW, and pinhole calibration ensured that the number of fluorescent molecules within the confocal plane (*N*) equaled 1.

### 2.6 Data processing: fitting a model

All raw data analysis was performed through SymPhoTime 64. After recording the fluctuations of the times versus fluorescence intensity trace, which indicated the diffusing fluorescent species in the detection volume, the autocorrelation function is applied and defined as:

$$G\;\left(\tau \right)=\frac{<I\;\left(t\right)\cdot I\;\left(t+\tau \right)>}{<I\;\left(t\right){>}^{2}}$$ (1)

The parameter I (t) represents the intensity time trace (Hz). The brackets indicate averaging over time. The autocorrelation time refers to the total duration during which the fluorescent species remains within the confocal plane.

For our standard measurements, the autocorrelation data was further fitted with a pure diffusion model:

$$G\;\left(t\right)=\sum_{i=0}^{n_{Diff}-1\;}\frac{\rho \left[i\right]}{\left[1+\frac{t}{\tau_{Diff}\left[i\right]}\right]\left[1+\frac{t}{\tau_{Diff}\left[i\right]k^{2}}\right]}$$ (2)

The parameter $\tau_{Diff}$ is the diffusion of the *i*th diffusing species in ms, $n_{Diff}$ represents the number of independently diffusing species, ρ represents the contribution of the *i*th diffusing species, and κ is the length of diameter ratio of the focal volume. This fitting model allows us to extract the diffusion time and number of molecules for each of our standard measurements, assuming only diffusion contributions are present in solution.

For our experimental data, GFP-OhyA plus lipid particles, the autocorrelation data was further fitted with a 3D-triplet kinetics model comprising 2 components:

$$\begin{array}{ll} G\;\left(t\right) & =\left[1+\sum_{j=0}^{n_{Trip}-1}T\;\left[j\right]\left[\exp\left(-\frac{t}{\tau_{Trip}\left[j\right]}\right)\right]\right]\\ & \;\sum_{i=0}^{n_{Diff}-1}\frac{\rho \left[i\right]}{\left[1+{\left[\frac{t}{\tau_{Diff}\left[i\right]}\right]}^{\alpha \left[i\right]}\right]{\left[1+\frac{{\left[\frac{t}{\tau_{Diff}\left[i\right]}\right]}^{\alpha \left[i\right]}}{\kappa^{2}}\right]}^{0.5}}+G_{\mathrm{Inf}} \end{array}$$ (3)

The parameter $\tau_{Trip}$ is the lifetime of the dark (triplet) state and $\eta_{Trip}$ is the number of dark (triplet) states. A triplet state refers to the non-fluorescent, long-lived excitation that causes a transition to the normal excited state. This event leads to a temporary “flickering” of fluorescence due to its dark nature. The presence of a triplet state can impact the shape of the ACF curve by causing a dip at the start of the curve. The anomaly parameter (α) of the *i*th diffusing species describes data points that significantly deviate from the expected pattern, thus enabling exclusion of these measurements when generating the model fit. $G_{\mathrm{Inf}}$ corresponds to the correlation offset. This fitting model describes diffusion of our species with triplet state blinking. This allows us to extract the diffusion time and number of molecules for each of our diffusing species by appropriately describing the triplet state and hindered diffusion.

We employed the single-molecule FCS technique to measure how vesicle addition affects protein diffusion, specifically by tracking the protein molecules rather than the vesicles themselves. The observed shift in protein diffusion time is caused by specific binding, as demonstrated by the side-by-side comparison of multiple lipid types (Section 3.2). The key readout from this experiment is the proportion of protein molecules that diffuse freely versus those with inhibited diffusion, which we interpret as the fraction of protein bound. It is important to note that we did not measure the number of protein molecules on each vesicle or the extent of the vesicle surface area covered.

### 2.7 Phosphorus-31 nuclear magnetic resonance (^31^P NMR) spectroscopy

DOPG (12 mM) was prepared in a buffer containing 150 mM NaCl, 1 mM bis-tris methane, pH 6.0, and 10% D₂O. GFP-OhyA was titrated into the DOPG solution at varying concentrations (3.9, 7.5, 16.7, 28.1, 42.9, 82.3, and 169.9 μM), using identical buffer conditions as the DOPG preparation.

^31^P NMR experiments were conducted at 298 K using a Bruker Avance 400 MHz spectrometer operating at 161.7 MHz. Proton decoupling was achieved with inverse-gated decoupling to minimize ^1^H–^31^P nuclear Overhauser effect (NOE) interactions. Data were collected using an observation frequency range of 65,789.48 Hz and a total of 64,000 data points. Acquisition parameters included a 90° pulse length and a 2 s pulse cycle. Approximately 22,000 accumulations were recorded before performing Fourier transformation of the free induction decay signal.

### 2.8 2D ^1^H–^13^C heteronuclear single quantum correlation (HSQC) NMR spectroscopy

Two-dimensional ^1^H-^13^C spectra were acquired to analyze interactions between DOPG and GFP-OhyA. DOPG (12 mM) was prepared in a buffer containing 150 mM NaCl, 1 mM bis-tris methane, pH 6.0, and 10% D_2_O, with and without 169.9 μM GFP-OhyA. Spectra were recorded at 298 K using a Bruker Avance 600 MHz NMR spectrometer equipped with four RF channels and a 5 mm z-gradient TXI CryoProbe (Bruker BioSpin), and operated with a ^13^C detection frequency of 150 MHz. This setup allowed for detailed analysis of ^1^H-^13^C correlations to investigate molecular interactions between the lipid and protein under defined conditions.

### 2.9 Statistical analysis

The χ^2^ values were used to assess the quality of fit for FCS data modeling in SymPhoTime 64. Statistical analyses and mathematical modeling of the processed data were conducted using GraphPad Prism software version 10.3.0.

The specific binding with Hill slope model is defined by the equation:

$$Y=\frac{B_{\max}*X^{h}}{K_{D}^{h}+X^{h}}$$ (4)

The binding model describes the relationship between ligand concentration (X) and specific binding (Y), with key parameters providing insights into the binding process. **X** represents the ligand concentration, while Y denotes the specific binding. The maximum binding capacity, **B**_**max**_, reflects the total number of available binding sites, and the dissociation constant, **K**_**D**_, indicates the ligand concentration required to achieve half-maximum binding at equilibrium, representing the binding affinity. The Hill slope **(h)** provides information about cooperativity: when **h = 1**, there is no cooperativity, meaning ligand binding at one site does not influence others. A Hill slope **h > 1** indicates positive cooperativity, where binding at one site enhances the likelihood of ligand binding at additional sites, while **h < 1** suggests negative cooperativity, where binding at one site reduces the affinity of other sites or indicates the presence of multiple binding sites with differing affinities.

## 3 Results

### 3.1 Biophysical properties of GFP-OhyA

An amino-terminal His-tagged version of GFP-OhyA (Figure 1A) was expressed in *Eschericia coli* and purified by Ni^+^ affinity and gel filtration chromatography to obtain a homogenous 97 kDa protein (Figure 1B). GFP-OhyA eluted predominantly as a single species on a Superose 6 Increase 10/300 GL column (Figure 1C). Like OhyA, the apparent molecular weight of GFP-OhyA estimated by its anisotropic light scattering pattern indicated the protein is 80.6% dimer in solution (Figure 1C). The 46.99°C ± 0.29°C melting temperature of GFP-OhyA (Figure 1D) indicates its structural resistance to thermal denaturation is within 1.31°C of OhyA (Radka et al., 2024). These data indicate GFP-OhyA is a stable protein with the correct OhyA quaternary structure.

### 3.2 Generating standard diffusion curves

**FIGURE 2:**
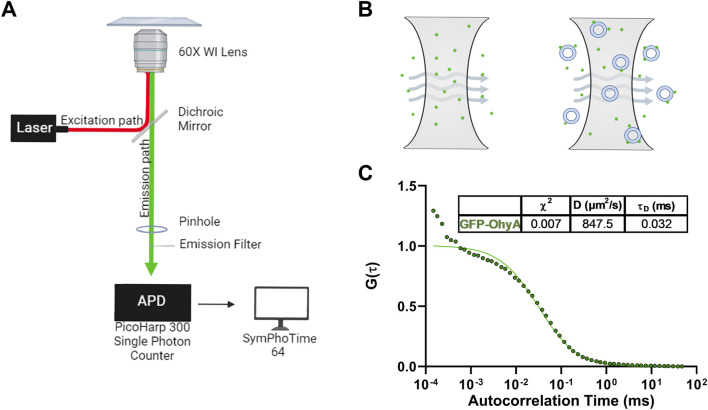
Microscope set-up and standard diffusion time measurements. **(A)**, Diagram of Nikon Eclipse Ti2 microscope set-up utilizing the confocal laser system. The Nikon Eclipse Ti2 microscope is connected to a PicoHarp300 single-photon counter which transmits the fluorescent signal to the SymPhoTime 64 software for analysis. **(B)**, Diagram of the illumination and detection volumes. Only fluorescent particles that diffuse through the confocal plane are detected by the photon counter for analysis. **(C)** *,* Fitted ACFs for freely diffusing GFP-OhyA protein in 50 mM KPO_4_, 150 mM NaCl, pH 6.0. A simple diffusion model (SymPhoTime 64) was utilized to fit the ACF and calculate diffusion parameters for the standards.

Fluorescence correlation spectroscopy (FCS) was used to monitor the binding of fluorescently labeled ligands to biomolecules (Figure 2A). Diffusion time (τ_D_), which scales with the hydrodynamic radius of diffusing molecules, was calculated from the autocorrelation of fluorescence time traces. We first compared the diffusion times of GFP-OhyA and Rhodamine PE-labelled large unilamellar vesicles (LUVs) and micelles. Fluorescence bursts in the 60 s time trace correspond to molecules passing through the confocal plane (Figure 2B). The intensity and duration of these bursts reflect fluorophore concentration and residence time in the detection volume.

**FIGURE 3:**
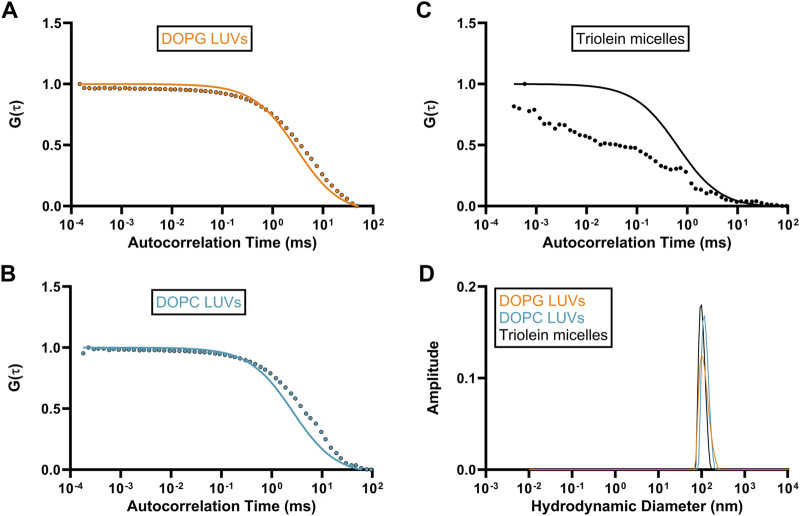
Standard diffusion time measurements for differing liposomal compositions. **(A–C)**, Fitted ACFs for freely diffusing DOPG LUVs, DOPC LUVs, and Triolein micelles constructed from 0.188 μM of accessible lipid in 50 mM KPO_4_, 150 mM NaCl, pH 6.0. A simple diffusion model (SymPhoTime 64) was utilized to fit the ACF and calculate diffusion parameters for the standards. **(D)**, Mass distribution curves for DOPG LUVs, DOPC LUVs, and Triolein micelles constructed from 3 μM of accessible lipid in 50 mM KPO_4_, 150 mM NaCl, pH 6.0. Measurements were collected via dynamic light scattering (DLS) analysis to confirm the size and homogeneity of each liposomal mixture.

Autocorrelation functions (ACF) derived from fluorescence time traces were used to determine diffusion times and molecular concentrations (Equations 1, 2). The best-fit normalized ACFs, G(τ), for GFP-OhyA is shown in Figure 2C, with a diffusion time of 0.032 ± 0.0012 ms. To evaluate lipid-binding preferences, we constructed LUVs of dioleoylphosphatidylglycerol (DOPG, anionic), dioleoylphosphatidylcholine (DOPC, cationic), and micelles of triolein (neutral lipid). Best fit ACFs for LUVs or micelles (Figures 3A–C; Table 1) exhibited longer diffusion times compared to free GFP-OhyA: 2.91 ± 0.31 ms for DOPG LUVs, 2.6 ± 0.27 ms for DOPC LUVs, and 0.65 ± 0.09 ms for triolein micelles. Dynamic light scattering measurements confirmed the lipid particles were monodisperse and of the expected size (Figure 3D). These results confirm that FCS effectively distinguishes free GFP-OhyA from lipid particle-bound protein based on shifts in diffusion time.

**TABLE 1:** Fitted autocorrelation parameters diffusion time (τ_D_), rate (D), reliability (χ^2^), and the mean hydrodynamic radius for lipid particle standards.

Lipid particle	χ^2^	D (μm^2^/s)	τ_D_ (ms)	Hydrodynamic radius (nm)
DOPG SUV	0.008	20 ± 2.2	1.3 ± 0.24	59.58 ± 1.19
DOPG + 18:1Δ9 SUV	0.011	27 ± 2.15	0.945 ± 0.09	58.74 ± 0.98
DOPG LUV	0.066	12.33 ± 1.32	2.91 ± 0.31	133.10 ± 2.10
DOPG + 18:1Δ9 LUV	0.05	9.97 ± 0.9	2.45 ± 0.245	130.90 ± 3.62
DOPC LUV	0.015	10 ± 1.1	2.6 ± 0.27	131.00 ± 3.37
Triolein micelle	0.118	42 ± 6.5	0.65 ± 0.09	113.00 ± 0.07

**FIGURE 4:**
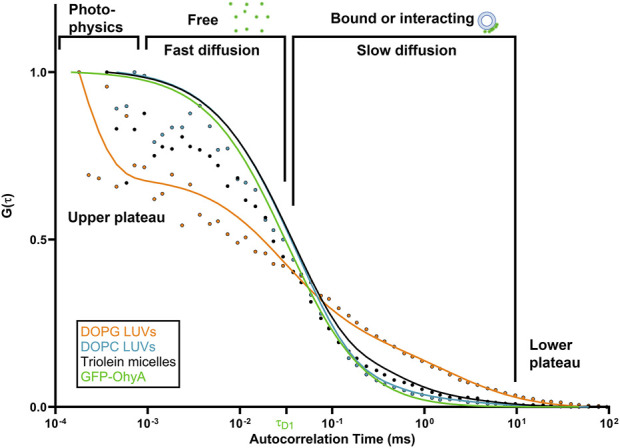
DOPG alters the diffusion behavior of GFP-OhyA via binding interactions. Representative normalized autocorrelation functions of 0.22 μM GFP-OhyA mixed with DOPG LUVs, DOPG + 18:1Δ9 LUVs, DOPC, and Triolein micelles constructed from 0.188 μM accessible lipids at 100 nm. Autocorrelation functions were fitted using a 3D-triplet kinetics model (SymPhoTime 64) comprising two components, utilizing a Gaussian 3D distribution. The diffusion time for free GFP-OhyA (τ_D_ = 0.032 ms) was held constant. Values for χ^2^, τ_D_, and D for each liposome composition and size are present in Table 1.

When GFP-OhyA binds its 18:1Δ9 (OA) substrate embedded in lipid membranes, its diffusion time increases–a hallmark of ligand binding to larger biomolecular complexes. Given the larger diameter of lipid particles relative to GFP-OhyA, we assumed that protein-loaded lipid particles diffuse with a similar coefficient as free particles. Mixing GFP-OhyA with lipid particles revealed a notable increase in diffusion time with DOPG LUVs but not DOPC LUVs or triolein micelles (Figure 4). Upon binding DOPG LUVs, GFP-OhyA diffusion time increased to 1.2 ± 0.12 ms and the ACF curve shape changed significantly Equation 3. Specifically, the upper plateau shifted downward, the lower plateau shifted rightward, and the slope connecting the plateaus broadened. These shape changes, combined with the shift to longer diffusion times, indicate that GFP-OhyA binds and interacts preferentially with anionic phospholipids.

### 3.3 Effect of lipid interactions on GFP-OhyA diffusion

**FIGURE 5:**
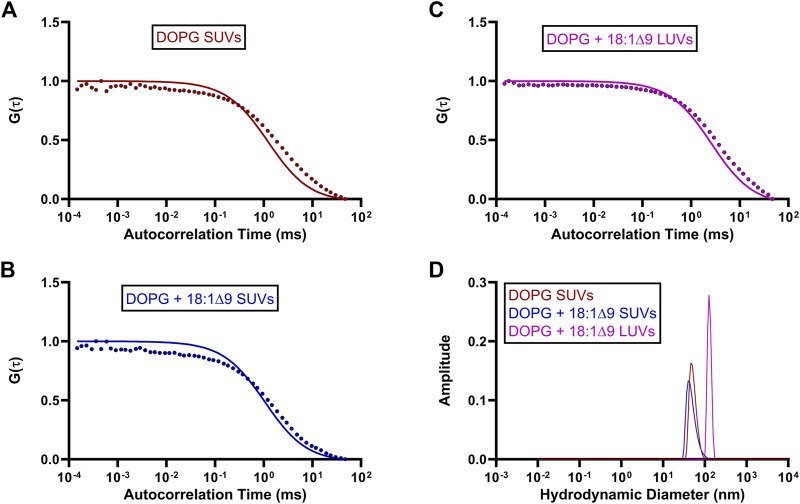
Standard diffusion time measurements of OhyA-specific lipid compositions. **(A–C)**, Fitted ACFs for freely diffusing DOPG SUVs, DOPG + 18:1Δ9 SUVs, and DOPG + 18:1Δ9 LUVs constructed from 0.188 μM accessible lipid in 50 mM KPO_4_, 150 mM NaCl, pH 6.0. A simple diffusion model (SymPhoTime 64) was utilized to fit the ACF and calculate diffusion parameters for the standards. **(D)**, Mass distribution curves for DOPG SUVs, DOPG + 18:1Δ9 SUVs, and DOPG + 18:1Δ9 LUVs constructed from 3 μM accessible lipid in 50 mM KPO_4_, 150 mM NaCl, pH 6.0. Measurements were collected via dynamic light scattering (DLS) analysis to confirm the size and homogeneity of each liposomal mixture.

**FIGURE 6:**
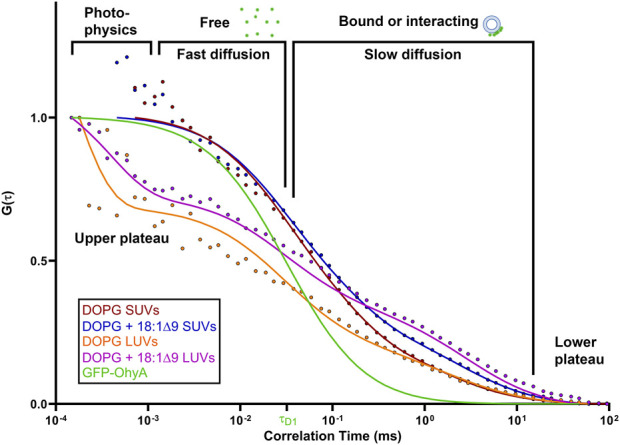
Protein•liposome binding alters the diffusion behavior of GFP-OhyA. Representative normalized autocorrelation functions of 0.22 μM GFP-OhyA mixed with DOPG SUVs, DOPG + 18:1Δ9 SUVs, DOPG LUVs, and DOPG + 18:1Δ9 LUVs constructed from 0.188 μM accessible lipids at 30 nm. Autocorrelation functions were fitted using a 3D-triplet kinetics model (SymPhoTime 64) comprising two components, utilizing a Gaussian 3D distribution. The diffusion time for free GFP-OhyA (τ_D_ = 0.032 ms) was held constant. Values for χ^2^, τ_D_, and D for each liposome composition and size are present in Table 1.

To investigate the effects of substrate availability and membrane curvature on GFP-OhyA binding, we prepared DOPG/OA LUVs and small unilamellar vesicles (SUVs) of DOPG and DOPG/OA (Figures 5A–D). Mixing GFP-OhyA with DOPG/OA LUVs increased its diffusion time to 2.6 ± 0.22 ms. With SUVs, diffusion times were 1.8 ± 0.42 ms for DOPG and 2.2 ± 0.14 ms for DOPG/OA Equation 3. While all DOPG-containing vesicles shifted the lower ACF plateau rightward and broadened the slope connecting the plateaus, only DOPG/OA LUVs caused a downward shift of the upper plateau (Figure 6). These findings indicate that GFP-OhyA interacts with all DOPG-containing vesicles, with larger LUVs exerting a more pronounced effect on the ACF curve than smaller SUVs. Although embedding OA substrates into vesicles had minimal impact on the substrate-free vesicle ACF curve’s shape, it consistently extended GFP-OhyA diffusion times.

### 3.4 GFP-OhyA concentration-dependent changes in bound fraction

**FIGURE 7:**
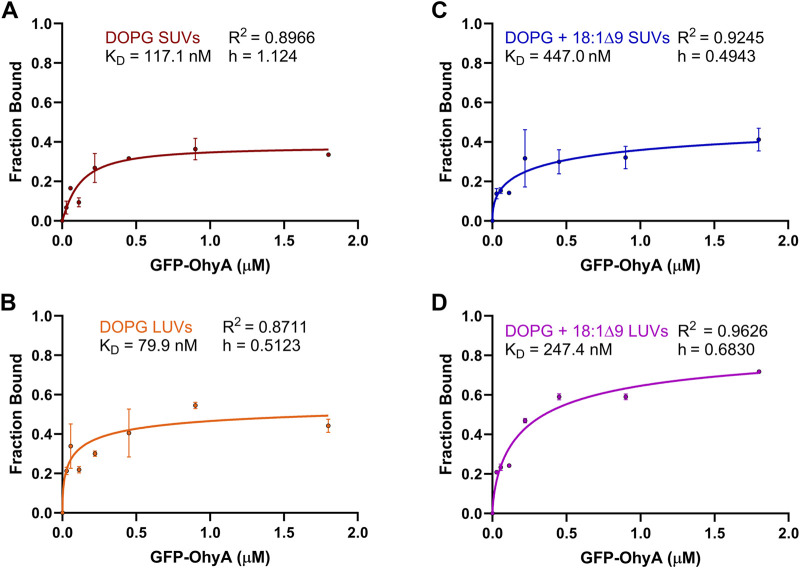
Fraction bound GFP-OhyA versus protein concentration. Fraction bound calculations were performed utilizing the autocorrelation function parameters ρ_1_ and ρ_2_ that describe the contribution of each diffusing species to the correlation function (bound GFP-OhyA vs. unbound GFP-OhyA). A constant accessible lipids concentration (0.188 μM) was used for protein titrations versus **(A)** DOPG LUVs, **(B)** DOPG SUVs, **(C)** DOPG + 18:1Δ9 LUVs, or **(D)** DOPG + 18:1Δ9 SUVs. Data are fitted to a specific binding with Hill slope model (GraphPad 10.3.0).

To further investigate the concentration-dependent binding of GFP-OhyA to vesicles, we used the SymPhoTime-derived ρ1 and ρ2 parameters from the fitted ACFs, reflecting the contributions of free and bound GFP-OhyA, respectively. The linear relationship between the GFP-OhyA concentrations used in the titration and those detected provides evidence that our measurements accurately correlate with the concentrations applied in each instance (Supplementary Figure S1). The fraction of GFP-OhyA bound was calculated and fitted to a specific binding with Hill slope model (Figure 7) (Equation 4). This binding model enables the extraction of the ligand concentration required to achieve half-maximum binding at equilibrium and fits a Hill slope to measure cooperativity. Results showed that substrate presence and lower membrane curvature influenced binding. At the highest GFP-OhyA concentration, the fraction bound to DOPG vesicles was 0.335 for SUVs (Figure 7A) and 0.442 for LUVs (Figure 7B). When DOPG/OA vesicles were used, these fractions increased to 0.412 for SUVs (Figure 7C) and 0.718 for LUVs (Figure 7D). Positive cooperativity was observed only when GFP-OhyA bound to substrate-free SUVs; in all other cases, the cooperativity was negative. These findings indicate that in both SUVs and LUVs, the presence of embedded substrate enhanced binding compared to substrate-free vesicles, despite reducing the binding affinity by 3- to 4-fold (from 79.9 to 247.4 nM for LUVs, and 117.1–447.0 nM for SUVs). The incomplete binding at high concentrations (fraction bound not reaching 1 asymptotically) suggests a reversible process with potential conformational states preventing complete vesicle saturation.

### 3.5 Impact of accessible lipid concentration on GFP-OhyA binding

**FIGURE 8:**
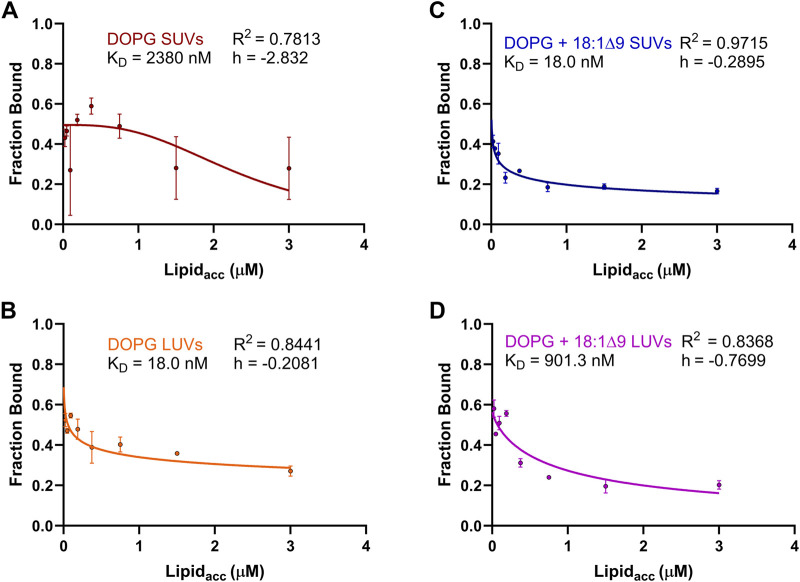
Fraction bound GFP-OhyA versus accessible lipid concentration (Lipid_acc_). Fraction bound calculations were performed utilizing the autocorrelation function parameters ρ_1_ and ρ_2_ that describe the contribution of each diffusing species to the correlation function (bound GFP-OhyA vs. unbound GFP-OhyA). A constant protein concentration (0.113 μM) was used for lipid titrations using **(A)** DOPG liposomes LUVs, **(B)** DOPG SUVs, **(C)** DOPG + 18:1Δ9 LUVs, or **(D)** DOPG + 18:1Δ9 SUVs. Data are fitted to a specific binding with Hill slope model (GraphPad 10.3.0).

The influence of accessible lipid concentration on GFP-OhyA binding was analyzed similarly to the concentration-dependent experiments. A specific binding with Hill slope model was used to fit the protein fraction bound (Figure 8). Interestingly, as lipid concentration increased, GFP-OhyA binding decreased. For DOPG vesicles, the SUVs initially increased in bound fraction before decreasing from 0.618 to 0.170 (Figure 8A), while the LUVs showed a gradual decline in bound fraction from 0.547 to 0.289 (Figure 8B). In DOPG/OA vesicles, the SUVs fraction bound decreased from 0.444 to 0.157 (Figure 8C), and the LUVs bound fraction decreased from 0.573 to 0.217 (Figure 8D). Negative cooperativity was observed across all vesicle types. These results suggest that higher lipid concentrations may impede GFP-OhyA binding.

### 3.6 Protein distribution on vesicles indicates different binding modes

**FIGURE 9:**
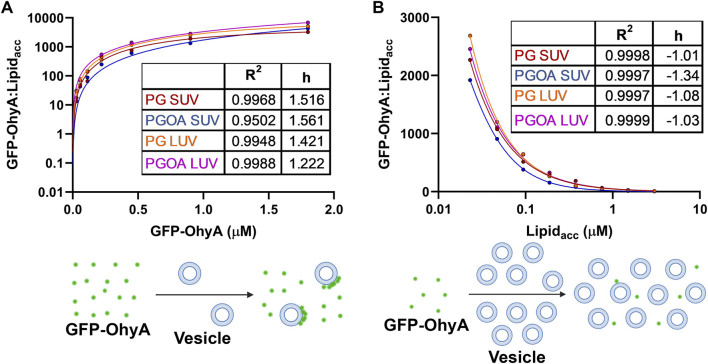
Ratio of bound GFP-OhyA (GFP-OhyA_mem_) to accessible lipid (Lipid_acc_) compared to total GFP-OhyA and accessible lipid titrations. **(A)**, The GFP-OhyA_mem_:Lipid_acc_ ratio versus GFP-OhyA data were fitted to a specific binding with Hill slope model. A representation of binding is displayed below, depicting interactions between liposome (blue) and GFP-OhyA (green) at a high GFP-OhyA concentration. **(B)**, GFP-OhyA _mem_:Lipid_acc_ versus Lipid_acc_ data were fitted to a specific binding with Hill slope model (GraphPad 10.3.0). A schematic for each binding mode is shown below each graph.

We normalized our results by calculating the ratio of bound GFP-OhyA to accessible lipid concentration to understand the role of protein distribution in membrane binding. As protein concentration increases with a fixed accessible lipid concentration, the ratio of vesicle-bound protein to accessible lipid concentration rises hyperbolically, demonstrating positive cooperative binding across all DOPG vesicle types (Figure 9A). This curve shape, with a distinct maximum, indicates that the number of vesicles carrying large quantities of protein increases relative to the protein molecules in solution. This suggests a protein:lipid molar ratio-dependent binding mode where protein molecules assemble on individual vesicles.

Conversely, increasing the lipid concentration while maintaining a constant protein concentration results is an exponential decline in the ratio of vesicle-bound protein to accessible lipid concentration, indicating negative cooperative binding across all DOPG vesicle types (Figure 9B). This curve, which exhibits a noticeable minimum, reflects a shift in the equilibrium between bound and unbound protein. As vesicle concentration increases, the protein-to-lipid molar ratio decreases, and the equilibrium shifts to a state with minimal protein bound, as the binding remains highly reversible. The apparent reduction in bound protein is not due to vesicles rendering bound protein undetectable by FCS, but rather to the equilibrium favoring the unbound state. This binding mode involves protein adsorption to the bilayer through electrostatic interactions, potentially disrupting protein assemblies or inhibiting their formation due to limited cooperativity or steric hindrance. Some protein molecules may bind to lipid sites in a way that excludes additional GFP-OhyA subunits from assembling, leaving unincorporated subunits unbound at fixed accessible lipid concentrations. This behavior underscores the dynamic and non-uniform nature of OhyA’s interaction with lipid membranes.

Notably, a membrane bilayer is essential for transitioning OhyA from discrete dimers to oligomeric ring assemblies that encircle vesicles (Radka et al., 2024; Oldham et al., 2024). The near-identical convergence of curves from both titration experiments indicates that OhyA’s association with the membrane is not significantly influenced by membrane curvature or substrate availability. Instead, protein-membrane binding is predominantly driven by protein:lipid molar ratio, reflecting the interplay between protein-protein and protein-lipid interactions.

### 3.7 Phosphorus (^31^P) NMR spectroscopy shows different binding modes

**FIGURE 10:**
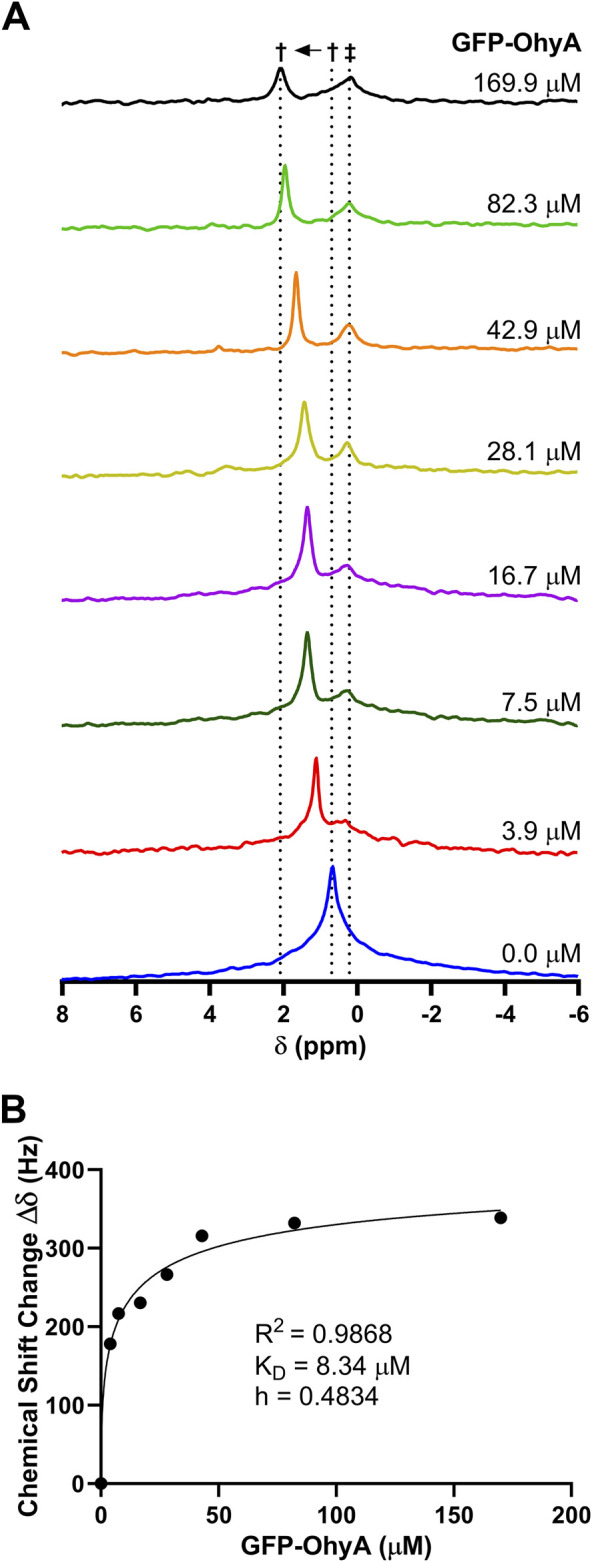
^31^P NMR spectrum of DOPG with GFP-OhyA. ^31^P NMR spectra were recorded as a function of increasing GFP-OhyA concentration. **(A)**, Stacked plot gives the ^31^P NMR spectra of free DOPG (0.68 ppm) and protein-bound DOPG (up to 2.14 ppm). Peaks marked with † are due to fast-exchange interaction between GFP-OhyA and DOPG, and peaks marked with ‡ are due to the intermediate-exchange interaction. **(B)**, Chemical shift data versus protein concentration from the fast-exchange binding interaction, fitted to a specific binding with Hill slope model (GraphPad 10.3.0).

Phosphorus-31 (^31^P) NMR spectroscopy was used to investigate the molecular interactions between GFP-OhyA and DOPG. Stacked NMR spectra (Figure 10A) revealed uniform and significant chemical shift changes (0.24 ppm) as the protein concentration increased, transitioning from the unbound state (0.68 ppm) to the bound state (2.14 ppm). These observations indicate fast-exchange binding of GFP-OhyA to DOPG that forms a slightly different size vesicle, characterized by a single sharp peak progressively shifting downfield with increasing protein concentrations.

At a protein concentration of 7.5 μM, a second smaller, broader peak appeared at 0.24 ppm, intensifying and broadening with increasing protein concentrations. This second peak, which shifts upfield of the unbound DOPG signal, remains at a constant chemical shift position and is consistent with intermediate-exchange binding with another vesicle of the same order.

The presence of both fast- and intermediate-exchange binding suggests two distinct binding events occur between GFP-OhyA and DOPG. At a protein-to-lipid concentration ratio slightly less than 1:50, the fast-exchange binding equilibrates, with no further chemical shift changes observed, while the intermediate-exchange peak intensity and broadening reaches its maximum. Two-dimensional ^1^H–^13^C HSQC NMR spectra were collected for DOPG LUVs in the presence and absence of GFP-OhyA. The hydrocarbon peaks of the lipids exhibited significant overlap in both conditions, indicating that the lipid structure and conformation remain unchanged upon protein binding (Supplementary Figure S2).

The dissociation constant (K_D_) for the fast-exchange binding was determined to be 3.181 ± 1.272 μM (Figure 10B). These results suggest that at low concentrations, GFP-OhyA transiently binds and releases from the membrane bilayer (fast exchange). In contrast, at higher protein concentrations, GFP-OhyA forms longer-lasting interactions on the bilayer as the peak intensity decreased, likely as part of an oligomeric complex.

### 3.8 Proposed model for OhyA oligomerization through conformational and membrane-binding dynamics

We propose that OhyA exhibits cooperative switching (Duke et al., 2001), where the molecules dynamically transition between individual molecules and oligomers in response to the concentration of nearby membrane-bound proteins. This transition is driven by conformational changes that promote oligomer formation through interactions with other OhyA molecules on the same membrane bilayer. Oligomer assembly is stabilized by membrane binding and destabilized by dissociation. OhyA stochastically switches between these states, with the likelihood of each state dependent on the local concentration of membrane-bound proteins. Membrane binding shifts the equilibrium toward the oligomeric state, as coupling energy from protein nucleation on the bilayer induces conformational changes propagated through allosteric interactions.

The occupancy of membrane-binding sites by individual molecules is a nonlinear function of protein concentration, as membrane affinity increases with the amount of bound protein (Stefan and Le Novere, 2013). Small changes in lipid concentration can disrupt oligomer assembly by increasing the fraction of unbound protein, and separating individual molecules. Under these conditions, transient oligomers form and rapidly dissociate. At higher concentrations of membrane-bound proteins, stable oligomers predominate. At intermediate concentrations, the system exhibits bistability, oscillating between oligomeric assemblies and dispersed molecules. This model underscores the critical roles of membrane binding and cooperative protein-protein interactions in regulating OhyA’s oligomeric switch, driven by conformation-dependent interactions between adjacent protomers.

## 4 Discussion

While the specific intermolecular interactions between OhyA and lipids that drive membrane binding have been previously identified, the equilibrium binding dynamics of OhyA in solution remain unexplored. Traditional binding models often assume a discrete stoichiometry and a Poisson distribution of particles that bind irreversibly, with unbound analyte levels rapidly declining as ligand concentration increases. However, modeling protein•membrane interactions poses a distinct challenge due to the multiple mechanisms influencing the collective biophysical properties of the interaction (White et al., 1998; Mulgrew-Nesbitt et al., 2006; Yeagle, 2014; Richens et al., 2015; Carravilla et al., 2020). Additionally, if binding is reversible, the unbound fraction cannot be ignored.

Using phosphorus-31 (^31^P) NMR spectroscopy, we observed two distinct binding events between GFP-OhyA and DOPG membranes. Fast-exchange binding, characterized by a significant chemical shift downfield of unbound DOPG, equilibrated at lower protein concentrations (K_D_ = 3.181 ± 1.272 μM). In contrast, intermediate-exchange binding, associated with a distinct upfield peak and significant line broadening, became prominent at higher protein concentrations. These results suggest that GFP-OhyA exhibits transient, reversible interactions with the membrane bilayer at low concentrations and forms more stable, long-lasting interactions at higher concentrations, likely as part of an oligomeric complex.

In this study, we explored how OhyA binds to membrane bilayers in a non-uniform manner, demonstrating that protein distribution on membranes differs significantly when driven by electrostatic attraction versus cooperative assembly into oligomeric complexes. Using fluorescence correlation spectroscopy (FCS), we measured the free diffusion of GFP-OhyA and lipid particles to study OhyA-membrane binding dynamics. Titrations of GFP-OhyA and accessible lipids revealed concentration-dependent variations in OhyA binding to vesicles. Increasing the lipid concentration while keeping the protein concentration constant revealed an exponential decay in the ratio of vesicle-bound protein to accessible lipid concentration, consistent with a dynamic shift in equilibrium. This reflects a transition from cooperative oligomeric binding at higher protein-to-lipid ratios to a fast-exchange binding regime at lower ratios, where the reversible nature of binding limits the fraction of bound protein. As vesicle concentration increases, protein molecules bind primarily through electrostatic interactions, with some sites on the bilayer restricting further assembly of GFP-OhyA subunits due to steric or cooperative constraints.

To further characterize this binding behavior, we analyzed OhyA interactions at high protein-to-lipid ratios. While the binding curves did not exhibit an overtly biphasic pattern, this model enabled the identification of two distinct binding events: an initial fast-exchange binding mode followed by an intermediate-exchange cooperative oligomerization. This two-state binding was observed using ^31^P NMR and substrate-free vesicles, offering additional insight into the interplay between protein-protein and protein-lipid interactions.

We also examined vesicles of different sizes and compositions to assess whether membrane curvature or substrate specificity influences OhyA binding. Our findings revealed that OhyA exhibits a moderate preference for membranes with lower curvature, where the presence of unsaturated fatty acid substrates slightly enhanced the overall interaction. Notably, the fraction of bound molecules did not asymptotically approach one, likely due to the combined effects of reversible association, steric hindrance, and a dynamic turnover rate governing the interaction. Together, these results underscore the complexity of OhyA-membrane binding and highlight the importance of molar ratios and cooperative mechanisms in regulating protein assembly on lipid bilayers.

OhyA is enriched in *S. aureus* extracellular vesicles (EVs) formed in response to antimicrobial fatty acids (Kengmo Tchoupa and Peschel, 2020). Our findings indicate that increased membrane curvature has a modest impact on OhyA binding, implying that the curvature associated with vesiculation does not actively recruit OhyA into EVs. GFP-OhyA localization studies in *S. aureus* cells confirm its membrane association (Radka et al., 2024), suggesting that OhyA’s presence in EVs is likely due to its pre-existing membrane localization rather than active recruitment during vesicle formation.

OhyA requires soluble flavin adenine dinucleotide (FAD) for catalysis (Subramanian et al., 2019; Radka et al., 2021b), and its transient, less favorable interactions with curved vesicle membranes may enable brief excursions into the vesicular cytosol to acquire FAD. Although the impact of FAD binding on OhyA-membrane interactions is unclear, there is evidence of communication between protein domains, with conformational changes in the membrane binding domain occurring in tandem with alterations in the FAD lobe (Oldham et al., 2024; Radka et al., 2024).

In this study, we explored how lipids affect the cooperative biophysical properties of OhyA-membrane binding. Our results, including insights from ^31^P NMR, provide a dynamic perspective on the dual role of lipids as structural stabilizers for single peripheral membrane protein binding and as facilitators of protein complex assembly. We conclude that mole fraction is the primary driver of OhyA•membrane association.

## Data Availability

The original contributions presented in the study are included in the article/Supplementary Material, further inquiries can be directed to the corresponding author.

## Abbreviations

OhyA, oleate hydratase; FCS, fluorescence correlation spectroscopy; DOPG, dioleoylphosphatidylglycerol; DOPC, dioleoylphosphatidylcholine; SUV, small unilamellar vesicle; LUV, large unilamellar vesicle; NMR, nuclear magnetic resonance.
