# Supplementary material for: Dissecting the biophysical mechanisms of oleate hydratase association with membranes
Source: Front Mol Biosci. 2025 Jan 8;11:1504373. doi: 10.3389/fmolb.2024.1504373 (PMC11751051; doi:10.3389/fmolb.2024.1504373)
Supplement: Supplementary file 1 [file DataSheet1.pdf]

Supplemental Figures

Dissecting the biophysical mechanisms of oleate hydratase association with membranes

William A. Lathram, Robert J. Neff, Ashley N. Zalla, James D. Brien, Vivekanandan Subramanian, Christopher D. Radka

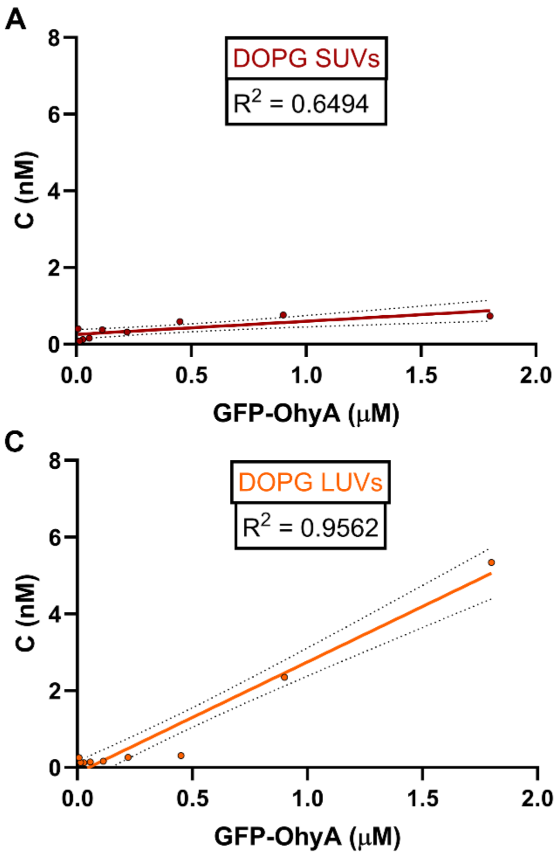

Figure S1. Validation of GFP-OhyA concentration within the confocal plane. Linearity of the calculated autocorrelation function parameter C, concentration of fluorescent molecules within the focal volume, versus the concentration of GFP-OhyA in the protein titration experiments. C is estimated by identifying the average number of fluorescently labeled particles within the detection volume. Points are fitted with a linear regression (GraphPad 10.3.0), with the 90% confidence interval shown by dotted lines.

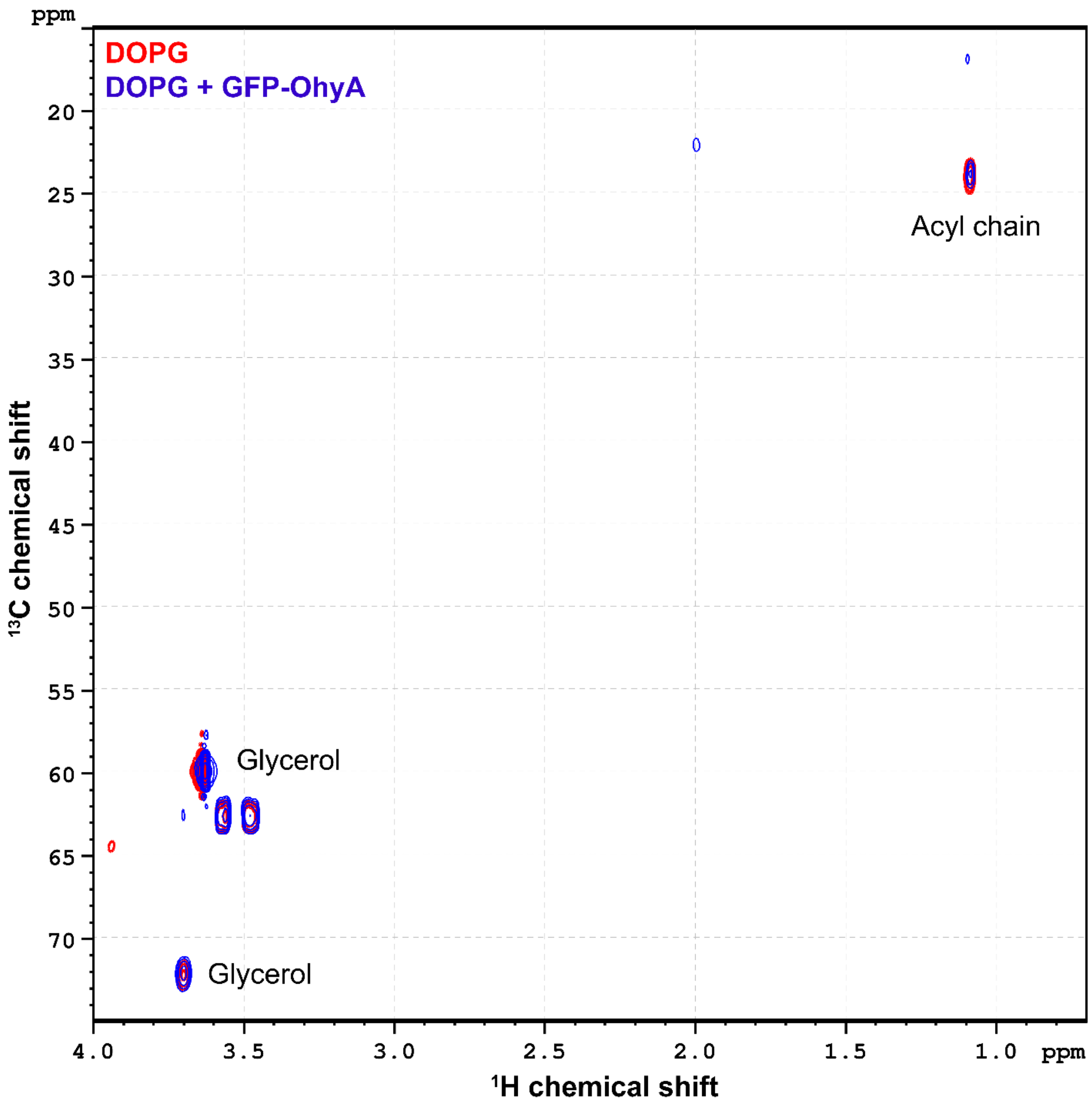

Figure S2. 2D ^{1}H–^{13}C HSQC of DOPG ± GFP-OhyA. Overlay of 12 mM DOPG LUVs in the absence (red) or presence of 169.9 μM GFP-OhyA (blue).
